# 5-Hydroxytryptamine (5-HT) Positively Regulates Pigmentation via Inducing Melanoblast Specification and Melanin Synthesis in Zebrafish Embryos

**DOI:** 10.3390/biom10091344

**Published:** 2020-09-19

**Authors:** Li Liu, Min Zhong, Jing Dong, Minghan Chen, Jing Shang, Yunyun Yue

**Affiliations:** 1 School of Traditional Chinese Pharmacy, China Pharmaceutical University, Nanjing 210009, Jiangsu, China; lilcpu@hotmail.com (L.L.); zhongmin18111@163.com (M.Z.); dongjingtx@gmail.com (J.D.); 13859874612@163.com (M.C.); 2 State Key Laboratory of Natural Medicines, China Pharmaceutical University, Nanjing 210009, Jiangsu, China; 3 Jiangsu Key Laboratory of TCM Evaluation and Translational Research, China Pharmaceutical University, Nanjing 210009, Jiangsu, China

**Keywords:** 5-hydroxytryptamine, zebrafish, melanocytes differentiation, melanocytes regeneration, protein kinase A signaling

## Abstract

It has been reported that 5-hydroxytryptamine (5-HT) is related to melanogenesis in mice and melanoma cells. However, the underlying mechanisms of 5-HT in regulating pigmentation remains unknown. In this study, we aim to clarify the regulatory mechanism of 5-HT in the pigmentation of zebrafish embryos and B16F10 cells. Our results show that 5-HT induces the pigmentation of zebrafish embryos in a dosage-dependent manner at concentrations of 0.01–1 mM. Whole mount in situ hybridizations and qRT-PCR in zebrafish embryos indicate that the expression of neural crest cells marker gene *sox10* is not changed in embryos treated with 5-HT compared to control group. The expression of *mitfa*, the marker gene of melanoblasts, is increased in the presence of 5-HT. Furthermore, 5-HT increased the expression of regeneration associated genes, namely *kita*, *mitfa*, and *dct,* after ablation of the melanogenic cells in zebrafish embryos. The experiments in B16F10 cells show that 5-HT promotes melanin synthesis by up-regulating the expression of key proteins MITF, TYR, TRP-1, and TRP-2. Especially, the small molecule inhibitor of PKA signaling, but not AKT and MAPK signaling, attenuates the up-regulation of MITF and TYR resulted from 5-HT induction in B16F10 cells. These results will help us to further understand the regulatory network of vertebrate pigmentation.

## 1. Introduction

Melanin, synthesized in vertebrates by a specific type of cell called melanocytes, is the main component of human hair, eyes, and skin color. The melanin distributed in the skin keratinocyte can protect human skin from UV damage [1,2]. Abnormalities of melanin synthesis, metabolism, and melanocytes function in human can lead to related skin diseases, such as albinism, vitiligo caused by insufficient pigment synthesis or loss of pigment, freckles, senile plaques, and even melanoma caused by abnormal accumulation of pigment. There is currently no highly effective treatment for these skin diseases. Therefore, it is of great significance to study the molecular mechanism of regulating pigmentation and to look for drugs regulating or metabolizing pigmentation to understand and treat skin diseases caused by abnormal pigmentation.

Melanocytes are derived from pluripotent neural crest cells and have the same developmental origin as peripheral nervous system [3,4]. The process of early melanocytes differentiation is similar among vertebrates. Neural crest cells specialized by transcription factor SOX10 have the potential to differentiate into melanoblasts, the pigment precursor cells [5]. The key transcription factor MITF (microphthalmia-associated transcription factor) expressed in melanocytes precursor cells can activate the expression of other melanocytes differentiation and migration related proteins, such as TYR (tyrosinase), DCT (dopachrome tautomerase, also known as tyrosinase-related protein 2), and TRP1 (tyrosinase-related protein 1) [6]. Subsequently, following the expression of MITF, TYR, DCT, and TRP1, melanoblasts gradually differentiate into melanocytes. After the formation of melanocytes, melanin synthesis is regulated by melanogenic enzymes which contain three major proteins: tyrosinase and tyrosinase-related proteins 1 and 2 [7].

Zebrafish (*Danio rerio*) is a small tropical fish and easily investigated the melanocytes function, because of its small size, transparent embryos and physiological similarity to mammals [8]. The pattern of zebrafish is generated by three type of pigment cells (melanophores, iridophores, xanthophores) [9]. There are different regulatory mechanisms of melanocytes development in larval and adult zebrafish. Similar to mammalian, the pigment cells are derived from the neural crest in zebrafish at the embryonic stage. Zebrafish skin melanocytes appear at approximately 24 h post-fertilization (24 hpf). The larval pigment pattern is established at approximately 60 hpf [10]. The mRNA of tyrosinase coding gene, *tyr* could be detected earliest at approximately 7 hpf. At 21 hpf, tyrosinase enzymatic activity can be detected [11]. Similar to mammals, the melanocytes of zebrafish possess the ability of regeneration [12]. The presence of melanocyte stem cells was suggested by observations of the unlimited capacity of melanocytes pattern re-establishment in the regenerating caudal fin [13]. Lots of signals are involved in the processes of melanocytes development, melanin synthesis, and melanocytes regeneration in zebrafish. *Sox10,* which encodes a transcription factor with C-terminal transcriptional activation domain and DNA-binding HMG domain, is critical for the formation and maintenance of neural crest cells. SCF/Kit signaling is essential for melanogenic lineage differentiation and regeneration. Kit signaling stimulates many signal transduction pathways, which is the key upstream signal to regulate melanoblast specification. It has been reported the role of Kit on the growth and differentiation of melanocytes. Zebrafish have two orthologues of mammalian Kit (*kita* and *kitb*) [14]. However, only *kita* is expressed in the melanophores. The larval melanocyte regeneration requires the kit receptor tyrosine kinase [15]. *Mitfa*, the homologue of mammalian Mitf, is the master regulator in specifying melanoblasts and inducing the expression of melanin synthesis associated key genes, such as *tyrp1a* and *dct*. However, it has been reported that *mitfa* is required at multiple stages of melanocyte differentiation, but not to establish the melanocyte stem cell in zebrafish. Usually, *mitfa* and *kita* are known as the early stage melanoblast markers, while *dct* and *tyr* are the differentiated melanocytes markers in zebrafish.

Biosynthesized from L-tryptophan, 5-hydroxytryptamine (5-HT) is a neurotransmitter, the actions of which are mediated via an interaction with certain receptors, including seven families (5-HTR1-7). It has been widely reported to play a key role in nervous system development and the regulation of memory, cognition, and mood. In recent years, some studies have indicated that 5-HT increased melanogenesis in three melanocytes lines (B16F10, SK-MEL-2, Melan-a) and mice [16]. Moreover, 5-HT receptors 1A/2A (HTR1A/2A) are involved in the regulation of 5-HT reuptake inhibitor, fluoxetine, in melanogenesis [17,18]. HTR1A binds NK1R (tachykinin receptor) to form protein heterodimer to negatively regulate melanogenesis in melanoma cells and mice [19]. Nevertheless, the regulatory mechanisms of 5-HT on pigmentation are also not clear.

In this study, we firstly examined the effect of 5-HT on zebrafish embryos melanocytes formation and regeneration. Secondly, we further investigated the downstream signal of 5-HT on promoting melanin synthesis in the B16F10 cell line. The results will help us to understand the regulatory mechanisms of 5-HT in regulating melanocytes development and melanin synthesis.

## 2. Materials and Methods

### 2.1. Zebrafish Maintenance and Ethic Statements

All zebrafish lines were purchased from China Zebrafish Resource Center (CZRC, China). All studies involving animal manipulations were approved by the institutional animal use and care committee of Nanjing Ruiying Runze Biopharmaceutical Technology Co., Inc. All animal experiments were performed in accordance with the National Institutes of Health Guidelines for the Care and Use of Laboratory Animals. Embryos were incubated at 28.5 °C and staged, according to the description by Kimmel et al. [20].

### 2.2. Drug Treatment

N-Phenylthiourea (PTU), 5-hydroxytryptamine, and 4-(4-morpholinobutylthio) phenol (MoTP) [21] were purchased from Sigma-Aldrich. MoTP was dissolved in dimethyl sulfoxide (DMSO) to make stock solution and then diluted in egg water to 50 µM for all treatments. PTU was dissolved in Milli Q water to make stock solutions and then diluted with egg water to 0.2 mM. Then, 5-HT was dissolved in Milli Q water to make stock solutions and then diluted with egg water to final treatment concentration.

### 2.3. Whole-Mount in situ Hybridization

Whole-mount antisense RNA in situ hybridizations were performed as described [22]. The riboprobes of *sox10*, *kita*, *mitfa*, and *dct* were previously described. The embryos were fixed in 4% paraformaldehyde (PFA) at 4 °C overnight, then bleached with H_2_O_2_. Digoxigenin-labeled antisense RNA probes were generated in vitro by using the zebrafish *sox10*, *kita*, *mitfa*, and *dct* cDNA as templates with RNA polymerase.

### 2.4. RNA Extraction and PCR

Total RNA was extracted using Trizol regent (Life Technology), and RT-PCR was performed in a two-step way with RT-PCR kit (Takara). Quantitative real-time PCR was performed with iQ SYBR Green Supermix (Takara) and analyzed with the IQ5 real-time PCR system (Bio-Rad). The expression of *sox10*, *mitfa*, and *dct* was assessed by quantitative PCR. Primers for those 3 genes are as follows: *sox10* (forward, 5′-GGTCACCATTGGGTGATGGA-3′, reverse, 5′-TCGCCTGATTTTCCTCCCTG-3′), *mitfa* (forward, 5′-GGGTTCATGG ATGCAGGACT-3′, reverse, 5′-TGAGGGGCAGGAGTTACTGA-3′), *dct* (forward,5′- GCACAGAACCACAGTTCAGC-3′, reverse, 5′-TGCGAGAAG TCGATGGCTTT-3′). All experiments were performed at least three times with similar results.

### 2.5. Measurement of the Pigmenting Activity in the Zebrafish

Synchronized embryos were collected, thirty embryos per well in 6-well plates, and cultured in 5 mL embryo medium. Then, 5-HT and MoTP were dissolved in 0.1% DMSO. In experiments, 0.2 mM PTU was administered from 6 to 35 hpf, then washed and bathed immediately in the medium with indicated concentrations of 5-HT at 35 hpf. The effects on the pigmentation of the zebrafish were observed under the stereomicroscope. All experiments were done at least three times with similar results.

### 2.6. Melanin Content and Tyrosinase Activity Assay

About thirty zebrafish embryos were washed with ice-cold PBS, and sonicated in lysis buffer at 4 °C. Then, lysates were centrifuged at 12,000 rpm for 10 min. Protein concentrations were determined by a BCA kit with bovine serum albumin (BSA) as a standard protocol (Beyotime Technology). Then, 100 μL supernatant containing the same 10 μg total protein was added to each well in a 96-well plate before being mixed with 100 μL 0.1% L-DOPA in 0.1 M PBS (pH 6.8) (M/V). After incubation at 37 °C for 0.5 h, the dopachrome was monitored by measuring the absorbance at 475 nm. Total melanin in the pellet was dissolved in 100 μL of 1 N NaOH with 10% DMSO for 2 h at 80 °C. Then, solubilized melanin was measured at 405 nm. Melanin content was calculated as a percent of the control. All experiments were done at least three times with similar results.

### 2.7. Cell Culture

The B16F10 melanoma cell line was obtained from the Cell Bank of the Chinese Academy of Sciences, Shanghai, China and cultured in Dulbecco’s Modified Eagle’s Medium (DMEM, Gibco) supplemented with 10% (v/v) fetal bovine serum (FBS, Gibco). Subsequently, 100 U/mL penicillin and 100 μg/mL streptomycin (Gibco, USA) were placed in a humidified incubator with 5% CO2 and 37 °C.

### 2.8. Western Blotting

Cells were washed with PBS and then dissolved in 100 μL lysis buffer for 20 min. After centrifugation at 12,000 rpm for 10 min, the protein suspension was obtained by collecting the liquid supernatant. Then, 30 μg proteins were loaded into a 10% SDS- PAGE gel before being transferred to PVDF membranes. The membranes were blocked with 0.25% albumin from bovine serum (BSA) for 1 h, then washed with tris-buffered saline (TBS) containing 0.1% Tween20 (TBST) three times, and incubated with MITF (ab12039), TYR (ab180753), TRP-1 (ab178676), TRP-2 (ab74073), p-CREB (Ser133, CST9198), CREB (CST9197), AKT (CST2920), p-AKT (CST4060), p-p38 (Thr180/Tyr182) (CST4631), p38 (CST9212), p-ERK1/2 (Thr202/Tyr204) (CST4376), ERK1/2 (CST4695), p-JNK (Thr183/Tyr185) (CST9251), JNK (CST9258), and β-actin (CST3700) with TBST. After the reaction with the second antibody, an enhanced chemiluminescence detection system was used to visualize the proteins. Densitometric analysis of the bands were performed using the ImageJ. Western blot results represented at least three independent experiments.

### 2.9. Statistical Analysis

All data are presented as mean ± SD. The statistical analyses of the results were performed with one-way ANOVA and *t* test, for comparing all pairs of columns, *p* < 0.05, 0.01, and 0.001 were accepted as statistically significant.

## 3. Results

### 3.1. 5-HT Induces Pigmentation in a Dose Dependent Manner in Zebrafish Embryos

**Figure 1:**
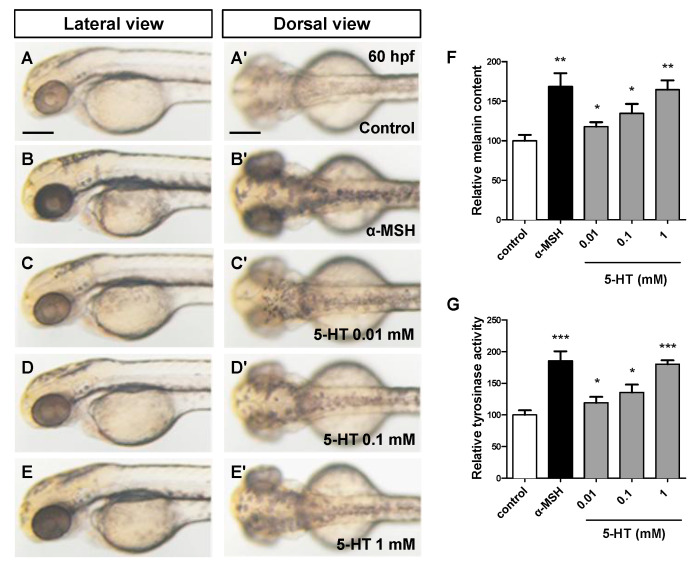
5-HT induces pigmentation in a dose dependent manner in zebrafish embryos. (**A**–**E**) Photos of zebrafish morphology at 60 hpf. The zebrafish embryos were pre-treated with 0.2 mM PTU from 9 to 35 hpf. a-MSH (**B**–**B’**) and different concentration 5-HT (**C**–**E’**) were added and incubated for a further 25 h (until 60 hpf). Every groups were showed at both lateral view (**A**–**E**) and dorsal view (**A’**–**E’**). (**F**) Relative melanin content was calculated through normalizing with control group. (**G**) Relative tyrosinase activity was calculated through normalizing with control group. * *p* < 0.05, ** *p* < 0.01, *** *p* < 0.001, compared vs. control. Error bars, S.D.

To investigate the effect of 5-HT on the pigmentation of zebrafish, a pre-treated PTU model (9–35 hpf) was used in this experiment to reduce pigment background. All the embryos were pretreated with PTU at 9 hpf, then washed and bathed immediately in the fresh egg water at 35 hpf. Different concentration 5-HT (0.01, 0.1, 1 mM) and α-MSH (positive control) (Figure 1B–B’) were added and incubated until 60 hpf. The results showed that, compared with control group (Figure 1A–A’), 5-HT significantly stimulated pigmentation of zebrafish eyes and trunk in a dose dependent manner (Figure 1C–E’).

Consistent with the result of melanin content (Figure 1F), the tyrosinase activity (Figure 1G) was also activated by 5-HT in a dose dependent manner. However, the increase of pigmentation could be resulted from the increase of the numbers of melanocytes or melanin synthesis. Therefore, we next examined the effect on melanocytes development of 5-HT in zebrafish embryos. Because of the most obvious pigmentation of 1 mM 5-HT, we chose this concentration for further study.

### 3.2. 5-HT Promotes the Processes of both Melanocytes Development and Melanin Synthesis

**Figure 2:**
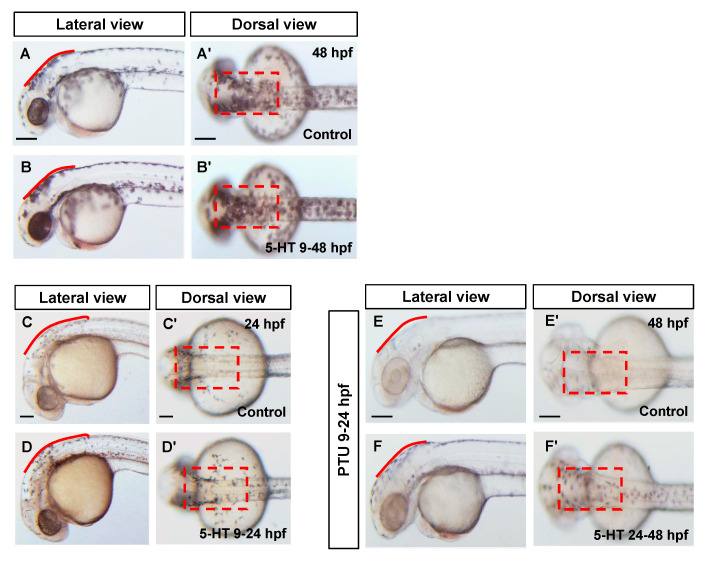
5-HT regulates both melanocytes development and melanin synthesis. (**A**–**B’**) Photos of zebrafish morphology at 48 hpf. Control group (**A**–**A’**) was not treated. The zebrafish embryos were treated with 1 mM 5-HT at 9 hpf and incubated until 48 hpf (B-B’). (**C**–**D’**) Photos of zebrafish morphology at 24 hpf. The embryos were treated with 1 mM 5-HT at 9 hpf and incubated until 24hpf (**D**–**D’**) compared with control group without treatment (**C**–**C’**). (**E**–**F’**) Photos of zebrafish morphology at 48 hpf. All zebrafish embryos were pre-treated with 0.2 mM PTU from 9 to 24 hpf. Then the zebrafish embryos were treated with 1 mM 5-HT at 24-48 hpf (**F**–**F’**) compared with control group without treatment (**E**–**E’**).

Melanocytes of zebrafish embryos, derived from neural crest cells, appear at approximately 24 hpf. Prior to that, the migratory neural crest cells move to the epidermis and differentiate into melanoblast from somitic stage in zebrafish embryos. Along with cell proliferation and differentiation, the melanoblasts express the master regulator Mitf and further induce the other key protein expressions to form functional melanocytes at about 24 hpf. To explore which stage of melanocytes pigmentation was affected by 5-HT, two time windows were examined. Firstly, we investigated the pigmentation effect of 5-HT (9–48 hpf) without treatment of PTU. Compared with control group, 5-HT significantly increases pigmentation of zebrafish embryos at 48 hpf (Figure 2A–B’). Then the zebrafish embryos were treated with 5-HT from 9 to 24 hpf, when the melanocytes differentiated from neural crest cells. We found that 5-HT increased the number of melanocytes and stimulated the pigmentation in zebrafish embryos at 24 hpf (Figure 2C,D’). These data indicated that 5-HT may play a positive role in regulating melanocytes development at early stage.

Next, zebrafish embryos were pre-treated with PTU from 9 hpf, then washed intensively and bathed immediately in the fresh egg water at 24 hpf. 5-HT were added and incubated for a further 24 h (until 48 hpf). Similarly, the pigmentation of zebrafish embryos was increased in the presence of 5-HT (Figure 2E–F’). It seems that 5-HT could promote both melanocytes development and melanin synthesis in zebrafish embryos.

### 3.3. 5-HT Facilitate the Melanoblast Specification from Neural Crest Cells

**Figure 3:**
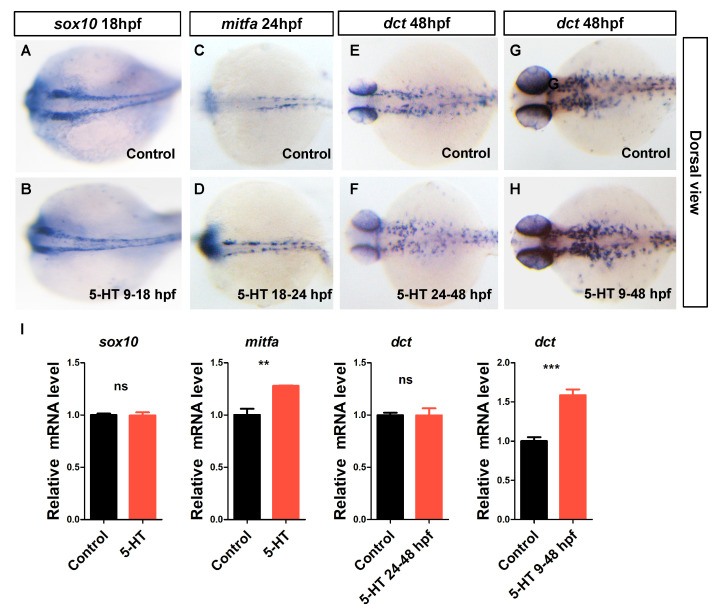
5-HT is sufficient to induce melanoblast specification from neural crest cells. (**A**-**B**) Whole mount in situ hybridization showed the expression of neural crest cells marker gene *sox10* at 18 hpf in zebrafish embryos of control group (**A**) and 5-HT treatment (1 mM, 9-18 hpf) group (**B**). (**C**,**D**) Whole mount in situ hybridization showed the expression of melanoblast marker gene *mitfa* at 24 hpf in zebrafish embryos of control group (**C**) and 5-HT treatment (1 mM, 18-24 hpf) group (**D**). (**E**–**H**) Whole mount in situ hybridization showed the expression of differentiated melanocytes marker gene *dct* at 48 hpf in zebrafish embryos. The embryos were treated with 5-HT from 24 to 48 hpf (**E**–**F**) and from 9 to 48 hpf (**G**–**H**). All photos were taken at dorsal view. (**I**) qRT-PCR results showed the relative expression level of *sox10* at 18 hpf, *mitfa* at 24 pf and *dct* at 48 hpf. The black column indicates the control group. The red column indicates the 5-HT treatment (1 mM) at different time window group. ns *p* > 0.05, ** *p* < 0.01, *** *p* < 0.001, compared vs. control. Error bars, S.D.

To clarify how 5-HT affect the melanocytes development in zebrafish embryos, whole mount in situ hybridization and quantative real time PCR were used to test the expression level of marker genes of neural crest cells (*sox10*), melanoblast (*mitfa*), and differentiated melanocytes (*dct*) [23]. To investigate whether the neural crest cells were affected by 5-HT, the expression of *sox10* was examined in presence of 5-HT from 9 to 18 hpf. The results showed that the expression of *sox10* was not changed in the zebrafish embryos treated with 5-HT (Figure 3A,B,I). This result indicated that 5-HT was not sufficient for the cell identity of neural crest cells. However, the expression of melanoblast marker gene, *mitfa* was increased in eyes and dorsal at 24 hpf with 5-HT treatment from 18 to 24 hpf (Figure 3C,D,I). That means 5-HT may play a key role in regulating neural crest differentiate into melanoblasts.

Interestingly, the expression of differentiated melanocytes marker gene, *dct* was not changed at 48 hpf with 5-HT treatment from 24 to 48 hpf (Figure 3E,F,I). However, the expression of *dct* was increased at 48 hpf when zebrafish embryos treated with 5-HT from 9 to 48 hpf (Figure 3G–I). Meanwhile, the number of *dct+* cells was not changed in the embryos treated with 5-HT or PTU (Figure 3G, Supplementary Figure S1). In summary, these results suggest that 5-HT plays a key role specifically in regulating melanoblast specification from neural crest cells but does not affect the cell identity of neural crest cells and the melanocytes differentiated from melanoblasts.

### 3.4. 5-HT Prompts Regeneration of Larval Zebrafish Melanocytes

Regeneration is one of the most popular concern of modern medicine. The regeneration of melanocytes brings hope to many skin diseases with melanocytes dysfunction or apoptosis, such as vitiligo. How to make the melanocytes cease to quiescent condition and reenter the cell cycle is the biggest challenge in melanocytes regeneration. It has been reported that the Kit receptor tyrosine kinase is essential for the regeneration of zebrafish larval melanocytes [24].

**Figure 4:**
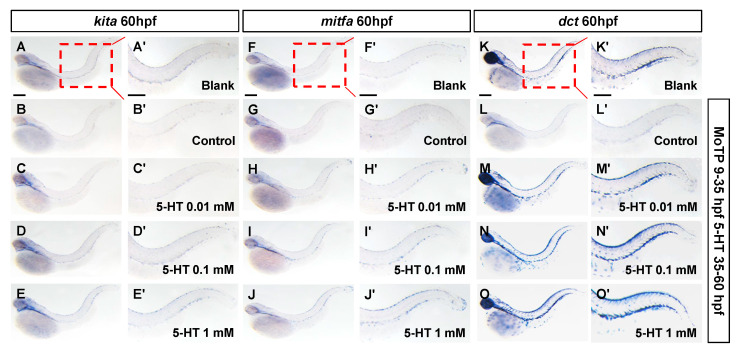
5-HT induces melanocytes regeneration in zebrafish embryos after ablation of melanogenic lineage by chemical. Whole mount in situ hybridization showed the expression of melanocytes regeneration related gene *kita* (**A**–**E’**), melanoblast marker gene *mitfa* (**F**–**J’**) and differentiated melanocytes marker gene *dct* (**K**–**O’**) at 60 hpf in zebrafish embryos. Blank groups were wild type zebrafish embryos without any treatment. Control groups were treated with 50 µM MoTP from 9 to 35 hpf to ablate most melanocytes and melanoblast cells. Then marker genes were tested at 60 hpf. The 5-HT groups were treated with 50 µM MoTP from 9 to 35 hpf, then treated with different concentration 5-HT (0.01, 0.1, 1 mM) from 35 to 60 hpf. The marker genes were tested at 60 hpf.

Since the 5-HT showed a promotion effect on melanogenic lineage, we speculated that whether 5-HT could induce the regeneration of melanocytes. In this study, the small molecule MoTP was used to ablate the melanocytes and melanoblast. The melanocytotoxicity was reported mediated via tyrosinase activity, presumably to convert MoTP to cytotoxic quinone species. The zebrafish embryos were pretreated with 50 µM MoTP from 9 to 35 hpf to eliminate most of the melanocytes at early stage. Compared to blank group, MoTP treatment resulted in decrease of melanoblast markers (*kita+* and *mitfa+*) and differentiated melanocytes (*dct+*) at 60 hpf in zebrafish embryos (Figure 4B,B’,G,G’,L,L’). When different concentrations of 5-HT were added into the medium of zebrafish embryos until 60 hpf. Surprisingly, we observed the increased expression of *kita* (Figure 4A–E), *mitfa* (Figure 4F–J), and *dct* (Figure 4K–O) by 5-HT induced in a dose dependent manner. The result manifests that 5-HT is sufficient for melanocytes regeneration in zebrafish embryos. However, the detailed mechanisms of 5-HT in promoting melanocytes regeneration still need further research.

### 3.5. 5-HT Increases the Expression of MITF and TYR by Activating the PKA/p-CREB Signaling in Melanoma Cells

Usually, 5-HT plays a role in different cells type through binding to the specific 5-HT receptor(s) on the cell membrane. Moreover, 5-HT receptors are a big G protein coupled receptor family, which include seven subfamilies from HTR1-7. The signaling transduction mediated by G protein involve cAMP/PKA, MAPK, DAG/PKC, and so on. At the start of this study, we found that 5-HT regulates not only melanocytes development, but also melanin synthesis. At the same time, the expression of differentiated melanocytes marker gene *dct* increased in 5-HT treated zebrafish embryos from 9 to 48 hpf without increase of melanocytes number, we surmised that 5-HT could regulate the melanin synthesis in differentiated melanocytes. Therefore, next, we want to clarify the key signaling pathway regulating melanin synthesis in the presence of 5-HT.

**Figure 5:**
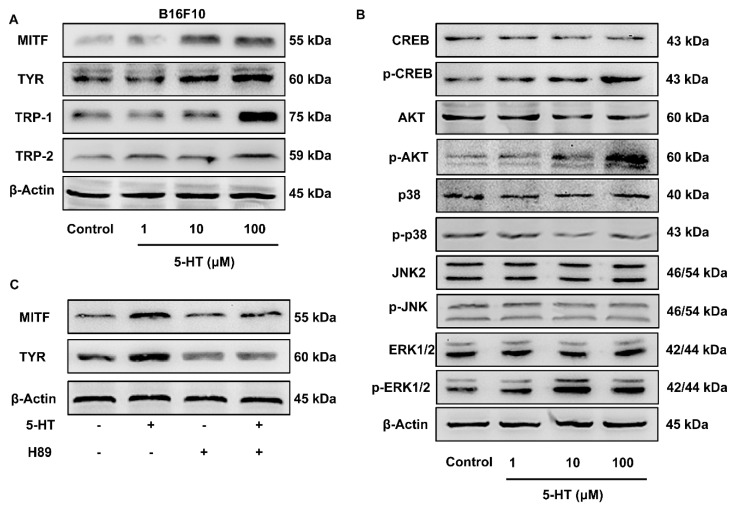
5-HT up-regulates MITF and TYR expression through activating PKA signaling pathway in B16F10 cells. (**A**) Western blot assays were performed to examine melanin synthesis related proteins, MITF, TYR, TRP-1 and TRP-2 in B16F10 cells with 5-HT treatment for 48 h. (**B**) Western blot assays show the protein levels of key signaling pathways, total CREB, phospho-CREB, total AKT, phospho-AKT, p38, phospho-p38, JNK2, phospho-JNK, ERK1/2, phospho-ERK. (**C**) Western blot assays show the expression of MITF and TYR in B16F10 cells treated with 5-HT and/or H89 (small molecule inhibitor of PKA signaling pathway). β-Actin was used for normalization. “+” means treat, “-” means no treat.

We examined the effect of 5-HT on the expression of key proteins related to melanogenesis, MITF, TYR, TRP1 and TRP-2 in melanocytes. The results showed that the expression levels of these four proteins were enhanced significantly at 48 h after 5-HT treated in B16F10 melanoma cells (Figure 5A, Supplementary Figure S2A–D). Furthermore, key proteins of PKA, AKT, and MAPK signaling pathways, p-CREB, p-AKT, p-p38, p-JNK, and p-ERK were tested by Western blotting. The results showed that p-CREB protein level was increased in the B16F10 cells with 5-HT treatment in a dose dependent manner (Figure 5B, Figure S2E). The inhibitor of PKA signaling, H89 showed an inhibiting effect on the expression increase of MITF and TYR with 5-HT treatment (Figure 5C, Supplementary Figure S2F,G).

Similarly, p-AKT and p-ERK protein levels were increased by 5-HT treatment in B16F10 melanoma cells (Figure 5B, Supplementary Figures S3A–S4A). This is consistent with the reality that 5-HT is a non-selective agonist of 5-HT receptors. However, the inhibitors of PI3K/AKT and ERK, LY294002 and PD98059, could not affect the increased expression of MITF and TYR by 5-HT treatment in B16F10 melanoma cells (Supplementary Figures S3 and S4).

In short, 5-HT may play a key role in promoting pigmentation through up-regulating the expression of melanogenesis related key proteins, MITF and TYR, by activating PKA/p-CREB specifically. However, which 5-HT receptor mediate this effect require further study.

## 4. Discussion

Despite the link between psychological stress and skin problems has long been well known, its specific association has never been fully clarified [25]. The function of those neurotransmitters in the physiological and pathological state of the skin remains poorly understood. Moreover, 5-HT is a phylogenetically ancient signaling molecule and neurotransmitter, which actions are mediated through 5-HT receptors [26,27]. It has been reported that 5-HT receptors are widely detected on mammalian dermal fibroblasts and melanocytes. Mammalian skin cells have the capability to produce and metabolize 5-HT. Further, 5-HT and its metabolite melatonin were associated with the regulation of numerous skin physiological functions [28,29].

In our previous study, the level of 5-HT in vitiligo patients’ serum was significantly decreased. Mice with chronic unpredictable mild stress exhibit hypopigmentation and a decrease of 5-HT level in both serum and skin. Fluoxetine, the classical 5-HT reuptake inhibitor, shows the ability to promote melanogenesis in melanoma cells and mice with psychological stress. All those studies indicate the crucial role of 5-HT in regulating the skin melanocytes maintenance and function. However, the underlying mechanisms have been rarely reported.

Mechanisms for specifying melanoblast from neural crest cells that involve upregulation of the microphthalmia transcription factor, Mitf (*mitfa* in zebrafish) in neural crest cells are conserved in mice, chick and zebrafish [30,31]. Transcription factor Sox10 is required for the expression of mitf in neural crest cells. When melanoblasts are specified, Mitf transcriptionally activates additional melanocytes differentiation and migration genes, including the melanin synthesis enzymes, dct, tyr, and trp1, and the melanocytes migration and survival gene, kit. Other signaling such as WNT and BMP are also involved in the melanocytes development [31,32]. Therefore, the effect on the cell fate determination of melanogenic lineage induced by 5-HT during embryonic stage and regeneration condition is what we want to illustrate in this research.

Firstly, 5-HT promoted pigmentation in a dose dependent manner in zebrafish embryos. Through further investigation, we found 5-HT had no effect on neural crest cells marked by transcription factor *sox10*, at 18 hpf in zebrafish embryos. However, the expression of melanoblast marker, *mitfa,* was increased at 24 hpf. When we treated the zebrafish embryos with 5-HT from 24 to 48 hpf, the expression of differentiated melanocytes marker gene *dct* was not affected. These results suggest 5-HT specifically play a key role in regulating melanoblast specification from neural crest cells. Secondly, we used small molecule MoTP to ablate the early melanoblast cells and differentiated melanocytes. After the ablation of melanogenic cells, the expression of essential genes for melanocytes regeneration, such as *kita* and *mitfa,* were increased with 5-HT treatment in zebrafish. The therapeutic method that pushes the resident progenitor cells to differentiate into melanogenic cells will greatly contribute to the treatment of skin diseases like vitiligo. However, the detailed role of 5-HT in regulating follicle stem cell cycle in mammals still needs to be elucidated.

Finally, we identified the downstream signaling activated by 5-HT in regulating melanin synthesis in differentiated melanocytes. B16F10 melanoma cell line is widely used for regulatory studies as an in vitro model. Here, we treated the B16F10 cells with 5-HT. The expression of the melanogenesis related key proteins, MITF and TYR were increased in the melanocytes with 5-HT treatment. Lots of signaling pathways, such as MAPK, PKA, AKT, and so on, have been reported to participate in the process of melanogenesis [33,34,35]. The MAPK signaling pathway, including p38, JNK, and ERK, is an important pathway involved in melanogenesis. Activations of the ERK and JNK pathways were related to the downregulation of melanogenesis [36,37]. P38 is involved in melanogenesis induced by UV irradiation. In this study, our data showed that 5-HT regulated the expression of MITF and TYR in B16F10 melanoma cells by specifically activating PKA/p-CREB signaling pathways. In terms of previous studies, HTR1A/2A are included in the regulation of melanogenesis. However, the crucial receptor of 5-HT in regulating melanocytes development and melanin synthesis needs to be identified.

## 5. Conclusions

In conclusion, our study showed that 5-HT induced the melanoblast specification from neural crest cells and regeneration in zebrafish embryos. In addition, 5-HT may prompt melanin synthesis by up-regulating the expression of melanogenic master regulator MITF and rate-limiting enzyme TYR. The protein kinase A signaling was further proven to be the key downstream signal of 5-HT in regulating melanogenesis in a melanoma cell line. These results will help to comprehensively understand the regulatory network of 5-HT in melanocytes development, regeneration, and function.

## Supplementary Materials

The following are available online at https://www.mdpi.com/2218-273X/10/9/1344/s1, Figure S1. 5-HT and PTU do not affect the number of *dct+* cells in zebrafish embryos. Figure S2. 5-HT up-regulate the expression of MITF and TYR by activating PKA/CREB signaling. Figure S3. 5-HT activates AKT signaling pathway in B16F10 cells. Figure S4. Effect of the 5-HT on MAPK signaling pathways in the B16F10 cells.
