# Supplementary material for: 5-Hydroxytryptamine (5-HT) Positively Regulates Pigmentation via Inducing Melanoblast Specification and Melanin Synthesis in Zebrafish Embryos
Source: Biomolecules. 2020 Sep 19;10(9):1344. doi: 10.3390/biom10091344 (PMC7563192; doi:10.3390/biom10091344)
Supplement: Supplementary file 1 [file biomolecules-10-01344-s001.pdf]

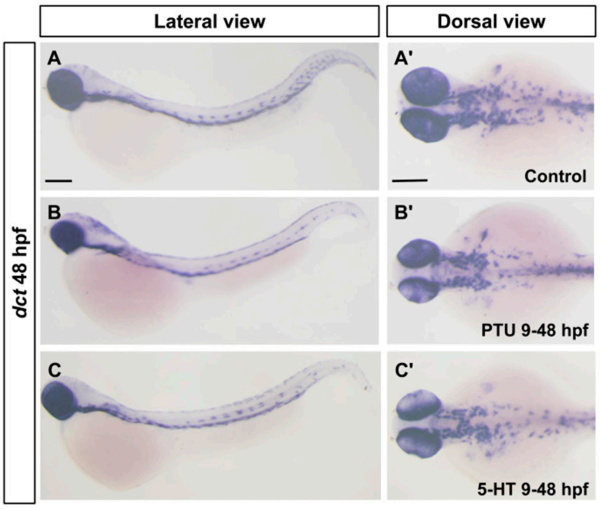

Figure S1. 5-HT and PTU do not affect the number of *dct*$^{+}$ cells in zebrafish embryos. (A-C') Whole mount *in situ* hybridization showed the expression of differentiated melanocytes marker gene *dct* at 48 hpf in zebrafish embryos of control group (A-A'), PTU treatment (0.2 mM, 9-48 hpf) (B-B') and 5-HT treatment (1 mM, 9-48 hpf) group (C-C').

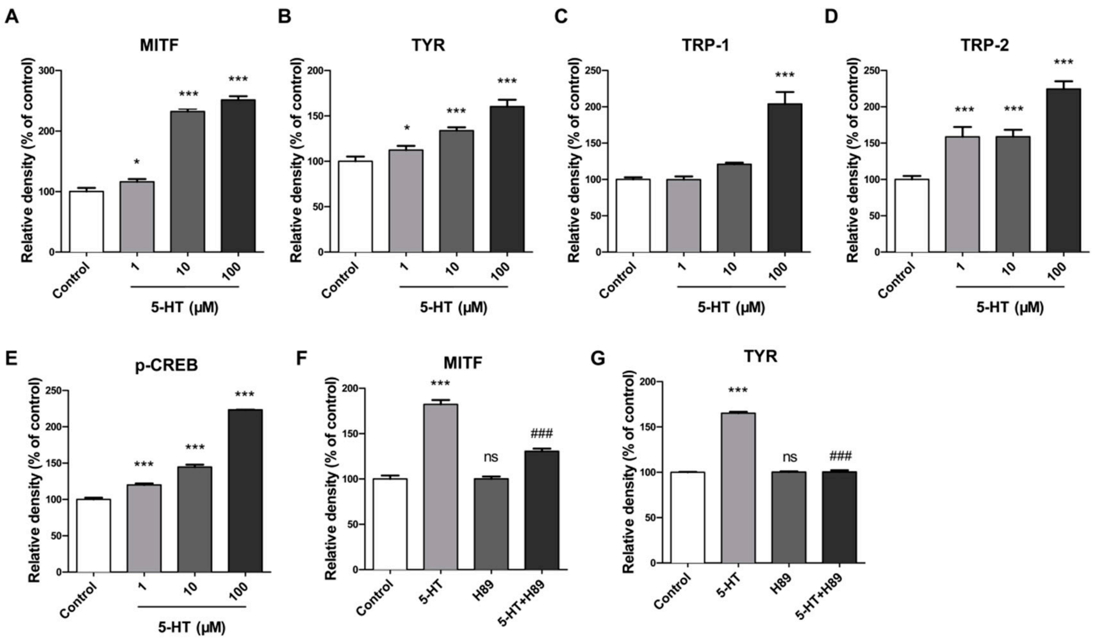

Figure S2. 5-HT up-regulate the expression of MITF and TYR by activating PKA/CREB signaling. Densitometry scanning of the band densities were utilized to measure the expression of proteins by Quantity One software. (A-D) Quantification of protein levels in Figure 5A. (E) Quantification of p-CREB protein level in Figure 5B. (F-G) Quantification of protein levels in Figure 5C. β-Actin were used for normalization. ns P>0.05, * P <0.05, *** P <0.001, compared vs control. ### P <0.001, compared vs 5-HT treatment group. Error bars, S.D.

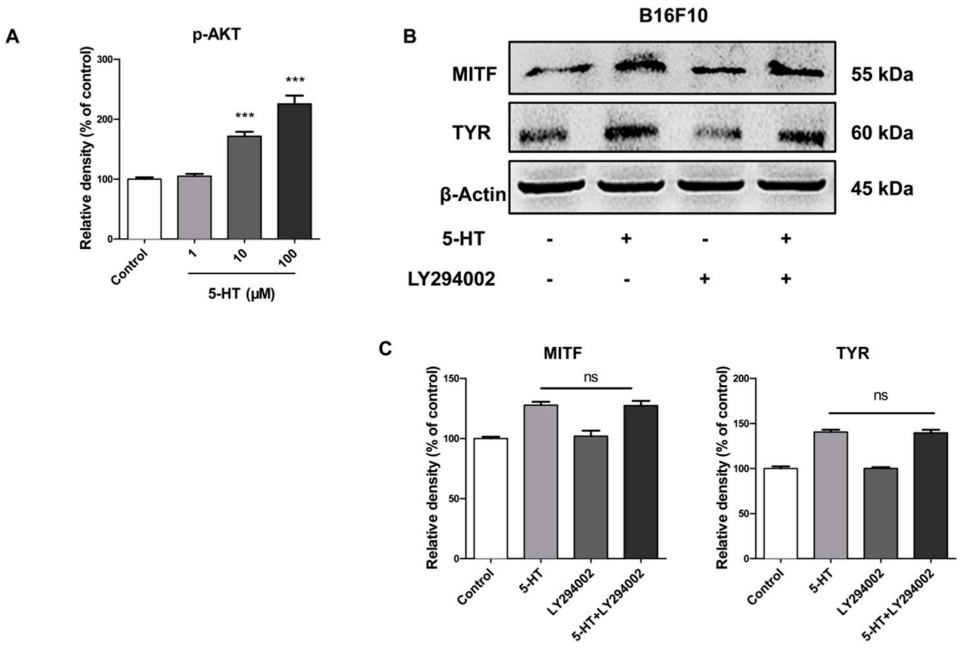

Figure S3. 5-HT activates AKT signaling pathway in B16F10 cells. (A) Quantification of p-AKT protein level in Figure 5B. (B) Western blot shows the effect of LY294002, the inhibitor of AKT signaling on 5-HT induced MITF and TYR expression in B16F10 cells. (C) Quantification of MITF and TYR protein level in Figure S3B. β-Actin were used for normalization. ns $P > 0.05$, compared vs 5-HT treatment group. Error bars, S.D.

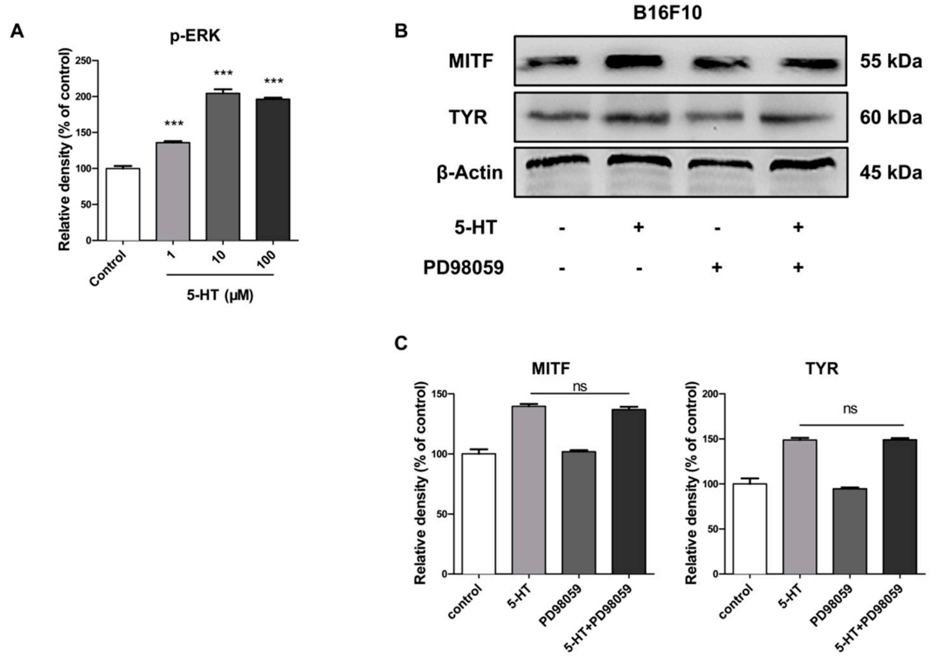

Figure S4. Effect of the 5-HT on MAPK signaling pathways in the B16F10 cells. (A) Quantification of p-ERK protein level in Figure 5B. (B) Western blot shows the effect of PD98059, the inhibitor of ERK signaling on 5-HT induced MITF and TYR expression in B16F10 cells. (C) Quantification of MITF and TYR protein level in Figure S4B. β-Actin were used for normalization. ns *P* >0.05, compared vs 5-HT treatment group. Error bars, S.D.
